# ^18^F-FDG silicon photomultiplier PET/CT: A pilot study comparing semi-quantitative measurements with standard PET/CT

**DOI:** 10.1371/journal.pone.0178936

**Published:** 2017-06-05

**Authors:** Lucia Baratto, Sonya Young Park, Negin Hatami, Guido Davidzon, Shyam Srinivas, Sanjiv Sam Gambhir, Andrei Iagaru

**Affiliations:** 1 Division of Nuclear Medicine and Molecular Imaging, Stanford University Medical Center, Stanford, California, United States of America; 2 Radiological Sciences Laboratory, Stanford University, Stanford, California, United States of America; 3 Departments of Radiology, Bioengineering, Materials Science and Engineering, Stanford University School of Medicine, Stanford, California, United States of America; Biomedical Research Foundation, UNITED STATES

## Abstract

**Purpose:**

To evaluate if the new Discovery Molecular Insights (DMI) PET/CT scanner provides equivalent results compared to the standard of care PET/CT scanners (GE Discovery 600 or GE Discovery 690) used in our clinic and to explore any possible differences in semi-quantitative measurements.

**Methods:**

The local Institutional Review Board approved the protocol and written informed consent was obtained from each patient. Between September and November 2016, 50 patients underwent a single ^18^F-FDG injection and two scans: the clinical standard PET/CT followed immediately by the DMI PET/CT scan. We measured SUV_max_ and SUV_mean_ of different background organs and up to four lesions per patient from data acquired using both scanners.

**Results:**

DMI PET/CT identified all the 107 lesions detected by standard PET/CT scanners, as well as additional 37 areas of focal increased ^18^F-FDG uptake. The SUV_max_ values for all 107 lesions ranged 1.2 to 14.6 (mean ± SD: 2.8 ± 2.8), higher on DMI PET/CT compared with standard of care PET/CT. The mean lesion:aortic arch SUV_max_ ratio and mean lesion:liver SUV_max_ ratio were 0.2–15.2 (mean ± SD: 3.2 ± 2.6) and 0.2–8.5 (mean ± SD: 1.9 ± 1.4) respectively, higher on DMI PET/CT than standard PET/CT. These differences were statistically significant (*P* value < 0.0001) and not correlated to the delay in acquisition of DMI PET data (*P* < 0.0001).

**Conclusions:**

Our study shows high performance of the new DMI PET/CT scanner. This may have a significant role in diagnosing and staging disease, as well as for assessing and monitoring responses to therapies.

## Introduction

Positron emission tomography (PET) plays a significant role in the diagnostic workup of cancer patients, evaluating various biological processes, including tumor cells metabolism. Indeed, PET has increasingly been recognized as a powerful tool for diagnosis, prognosis and determination of response to therapy in oncology [1].

2-deoxy-2-[fluorine-18]fluoro-D-glucose (^18^F-FDG), an analogue of glucose, is the most used PET radiotracer in clinical practice, providing information based on the increased glucose uptake and glycolysis of cancer cells [2]. Semi-quantitative measurements, such as maximum standardized uptake value (SUV_max_), are strongly correlated with the ^18^F-FDG metabolic rate and often used as a biomarker of cancer response to predict or assess the efficacy of treatments [3–5].

However, SUV_max_ measurements can be influenced by different factors, including patient’s characteristics (weight, plasma glucose level), time between injection and scan (uptake time) as well as data reconstruction methods [6–8].

Hybrid imaging systems such as PET/CT improved detectability of cancer lesions, providing both accurate anatomical locations and metabolism information [9]. Since its introduction in 2002, many technological improvements have been made both in terms of hardware and software for hybrid PET/CT scanners, leading to better imaging quality, higher spatial resolution and more accurate image reconstruction.

We recently installed a new generation PET/CT scanner (GE Discovery Molecular Insights—DMI PET/CT, GE Healthcare, Waukesha, WI). Here we evaluated if the DMI PET/CT scanner provides equivalent results compared to the standard of care PET/CT scanners (GE Discovery 600 or GE Discovery 690) used in our clinic and explored any possible differences in semi-quantitative measurements.

## Materials and methods

### Clinical study

The local Institutional Review Board (Stanford University Research Compliance Office) approved the protocol and written informed consent was obtained from each patient. Between September and November 2016 we enrolled 50 patients who had an indication for a clinical standard of care PET/CT exam and were more than 18 years of age. Women were included if not of reproductive age. We excluded diabetic patients with a glucose level ≥ 150 mg/dl at the time of the scan and those unable to tolerate the exam.

### Standard PET/CT protocol

All patients underwent a single injection dual imaging protocol starting with the standard of care PET/CT scan (GE Discovery 600 PET/CT scanner or GE Discovery 690 PET/CT scanner, as scheduled for routine oncological examination), followed immediately by image acquisition using the new DMI PET/CT scanner.

A dose of 370MBq (10 mCi) of ^18^F-FDG was injected (range: 8–11.6 mCi; mean ± SD: 9.6 ± 0.9). The participants fasted at least 6 hours prior to the scans and blood glucose levels were less than 150 mg/dl at the time of ^18^F-FDG injection. A “smart current” CT scan was obtained from the skull base to the mid-thighs and used for attenuation correction purposes and to help in anatomic localization of ^18^F FDG uptake. Immediately after the CT, an emission PET scan was acquired over the same anatomical regions. The acquisition time was 3 minutes per bed position (47 slices/bed) in 6 beds with 11-slice overlap at the edge of the axial field of view. PET images were reconstructed using ordered subset expectation maximization (OSEM) with 2 iterations and 32 subsets for Discovery 600 or 2 iterations and 24 subsets for Discovery 690, per vendor’s recommendations.

### DMI PET/CT protocol

Immediately after completion of the standard of care PET/CT scan, all patients underwent a second scan using the DMI PET/CT. The DMI is time-of-flight (TOF) enabled and uses detectors that combine lutetium-based scintillator crystal arrays with a silicon photomultiplier (SiPM) bloc design to improve sensitivity. CT was done with very low current (10 mA, 120 kV) and used for attention correction and anatomic localization. PET data were acquired over the same anatomical regions and using the same acquisition times as in standard PET/CT (3 minutes per bed). PET images were reconstructed using OSEM with 3 iterations, 16 subsets, 5mm post filter in a 256x256 matrix, as well as using block sequential regularized expectation maximization (BSREM, Q.Clear^®^) reconstruction (beta value of 400 in a 256 x 256 matrix) [10, 11].

### Imaging analysis

Images were reviewed and analyzed independently by two blinded readers (LB, with 6 years of experience, AI, with 11 years of experience) using Advantage Workstation (GE Healthcare). A circular 2 cm region-of-interest (ROI) was placed in up to four detected lesions (primary tumor and 3 additional FDG-avid lesions of different tissues) and in normal tissues (cerebellum, parotid gland, aortic arch, normal lung, liver, spleen, gluteal muscle and gluteal fat). All lesions identified on the initial scan were identified and matched to lesions on the subsequent scan. The ROIs for the liver and spleen were placed in areas of normal parenchyma. Maximum and mean standardized uptake values were recorded (SUV_max_—SUV_mean_); lesion-to-blood pool SUV_max_ and lesion-to-liver SUV_max_ were also used as relative measure of contrast. Measurements from the DMI PET/CT were collected on images reconstructed with both TOF and Q.Clear^®^, as well as without TOF and Q.Clear^®^.

### Statistical analysis

Statistical analysis was performed with SPSS v21 (SPSS Inc. Chicago, IL) and MedCalc v.15.8 (MedCalc Software BVBA, Ostend, Belgium). Continuous data are presented as mean ± SD, minimum-maximum values and frequencies (%) for categorical variables. Statistical comparison of SUV_max_ data for both scans was performed with a 2-tailed paired Student t test.

Bland-Altman plot was used to evaluate the degree of agreement between different scanners. We used Partial Correlation and Linear Regression analysis to evaluate the relationship between different SUV_max_ and time delay between scans. A *P*-value of <0.05 was considered significant. Equivalence test was performed on each of the background organ with an equivalence interval of [-0.7, 0.7].

## Results

Fifty patients were enrolled in the study. There were 44 men (88%) and 6 women (12%), 27–94-year-old (mean ± SD: 61 ± 15.6 years). Eighteen percent (9/50) of the participants were referred for initial treatment strategy (formerly diagnosis and initial staging), while eighty-two percent (41/50) of them were referred for subsequent treatment strategy (including treatment monitoring, restaging and detection of suspected recurrence), based on the National Coverage Determination for ^18^F FDG PET for Oncologic Conditions from the Centers for Medicare & Medicaid Services [12].

Standard of care PET/CT images were acquired 43.3–112.6 minutes (mean ± SD: 64.4 ± 13.5) after injection of 8–11.6 mCi (mean ± SD: 9.6 ± 0.9) of ^18^F FDG. DMI PET/CT scans started at 71.2–142.2 minutes (mean ± SD: 102.1 ± 17.4 minutes) after the ^18^F FDG injection. The delay time between start of standard of care PET/CT and DMI PET/CT was 17.5–75.1 (mean ± SD: 37.7 ± 13.9).

The results of equivalence tests, with an equivalence interval of [-0.7, 0.7], performed on each of the normal tissues matched per patient are presented in Table 1. Equivalence was met for all background tissues except for the cerebellum (*P* value < 0.05).

**Table 1:** Equivalence testing of baseline organs matched per patient per PET/CT scan type.

Baseline organs	Difference of SUV_mean_	90% CI	*P*	Equivalence level
**Parotid gland**	0.20±0.37	-0.398; -0.007	<0.0001	Equivalent
**Normal lung**	-0.12±0.25	0.050;0.190	<0.0001	Equivalent
**Spleen**	-0.18±0.33	0.052;0.303	<0.0001	Equivalent
**Gluteal muscle**	0.04±0.16	-0.112;0.037	<0.0001	Equivalent
**Gluteal fat**	-0.05±0.09	0.020;0.081	<0.0001	Equivalent
**Aortic arch**	-0.46±0.35	0.344;0.569	=0.0002	Equivalent
**Liver**	-0.48±0.40	0.340;0.624	<0.006	Equivalent
**Cerebellum**	0.23±1.66	-0.957;0.489	0.14	Not Equivalent

CI = Confidence interval.

**Fig 1:**
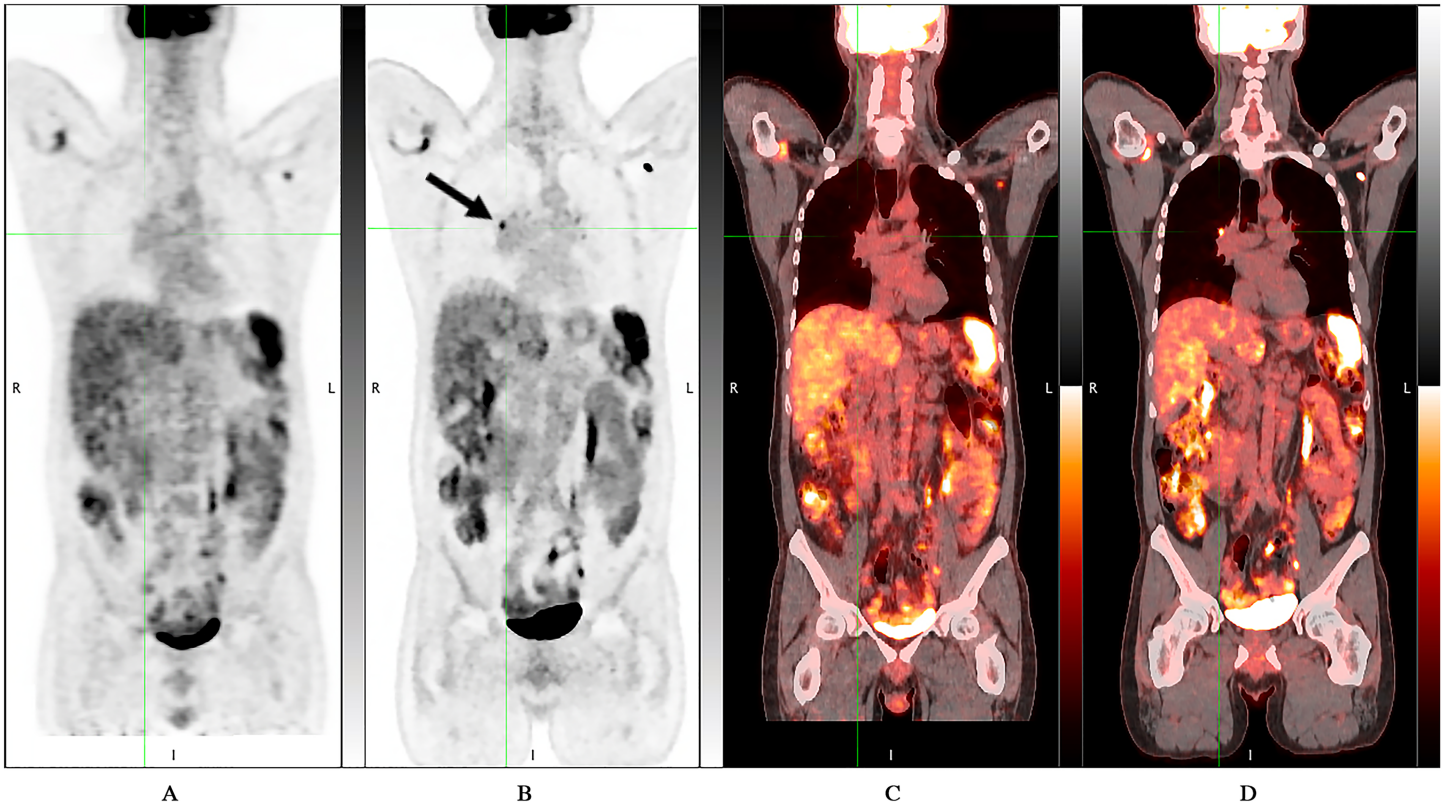
D690 PET/CT vs DMI PET/CT: DMI showed an additional focal avid FDG uptake area (black arrow). 46 years-old man with myocardial sarcoidosis who underwent PET/CT for initial staging. DMI images were acquired 39.1 minutes after standard acquisition and 109.2 minutes after ^18^F-FDG injection. A) D690 Maximum-intensity-projection (MIP); B) DMI MIP; C) D690 coronal fused image D) DMI coronal fused image.

**Fig 2:**
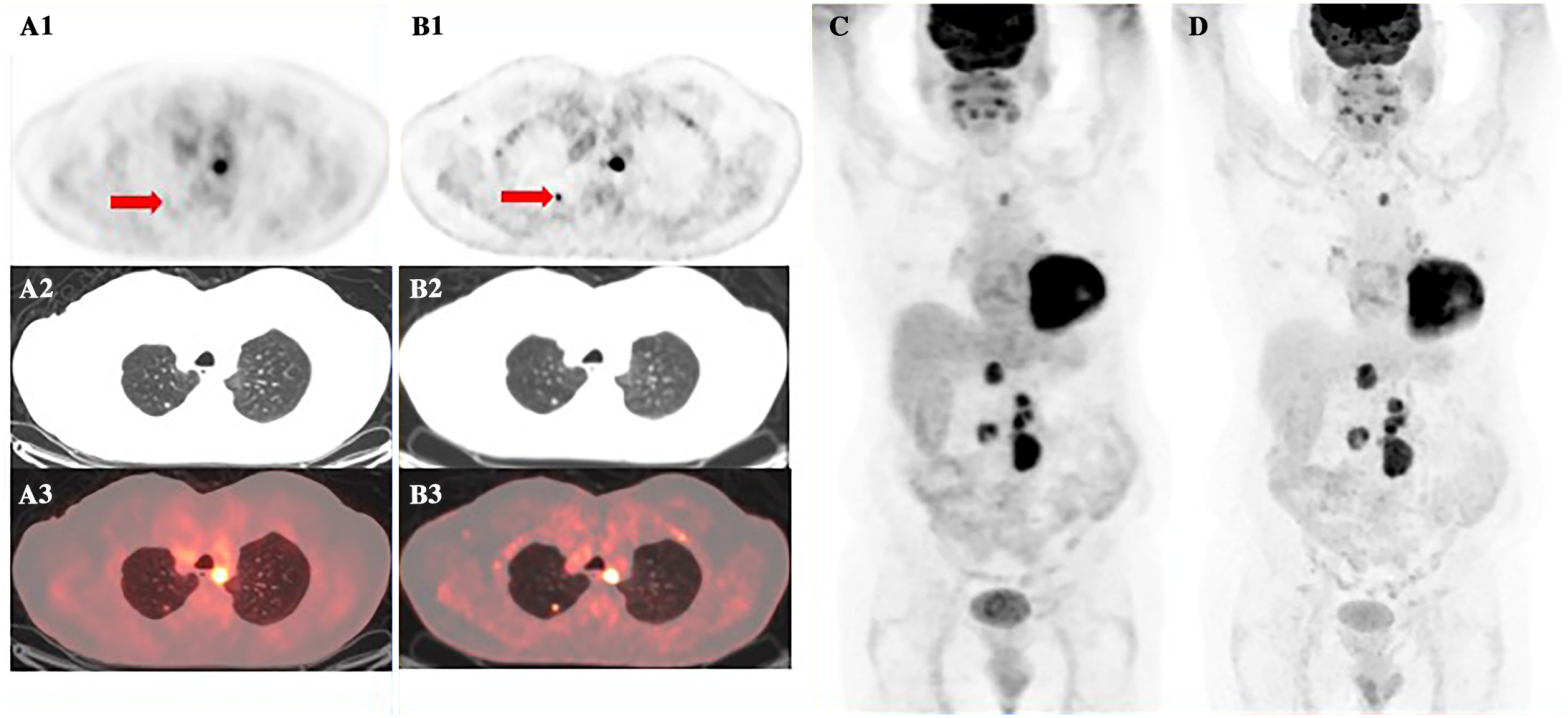
D690 PET/CT vs DMI PET/CT: DMI showed a significant increase in SUV_max_ (red arrow). 79 years-old man with kidney cancer who performed PET/CT for evaluation of response to therapy. DMI images were acquired 34 minutes after standard PET/CT acquisition and 104 minutes after ^18^F-FDG injection. A1) D690 PET; A2) D690 CT; A3) D690 fused image; B1) DMI PET; B2) DMI CT; B3) DMI fused image; C) D690 MIP; D) DMI MIP.

**Fig 3:**
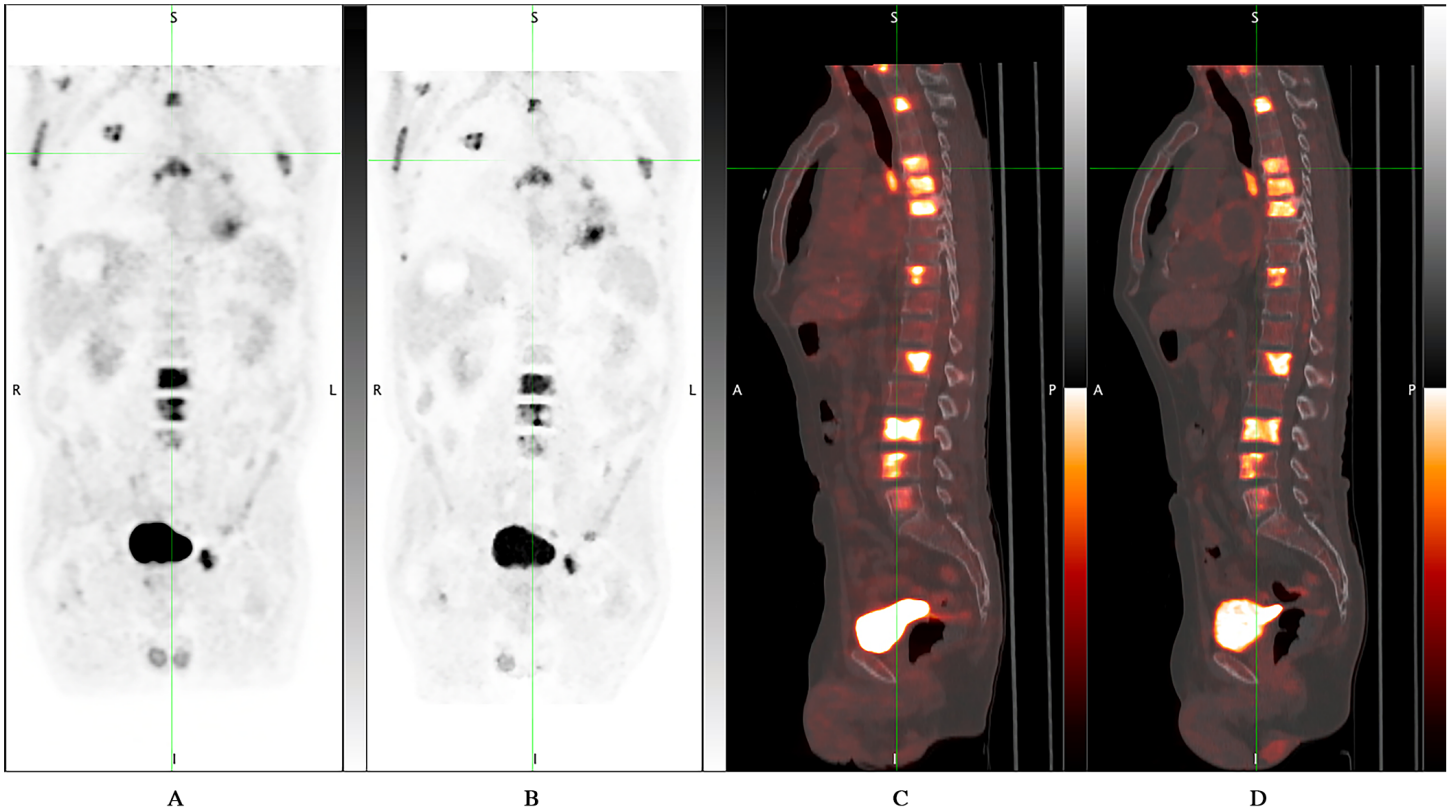
D600 PET/CT vs DMI PET/CT: no additional bone lesions were detected by DMI PET/CT compared to standard PET/CT. 61 years-old man with tonsillar cancer who underwent PET/CT for evaluation of response to therapy. DMI images were acquired 47.4 minutes after standard acquisition and 126 minutes after ^18^F-FDG injection. A) D600 MIP; B) DMI MIP; C) D600 sagittal fused image; D) DMI sagittal fused image.

On a per patient analysis, 12 of the 50 patients had no abnormal uptake on either standard of care PET/CT or DMI PET/CT. DMI PET/CT identified all the lesions seen using the standard of care PET/CT, as well as additional 37 FDG-avid lesions in 14 of 50 patients (28%). DMI PET/CT identified more lymph nodes than standard of care PET/CT in 12 patients. A similar finding was noted for lung nodules in 5 patients, liver lesions in 2 patients and other areas of additional focal uptake in 1 patient. Same number of osseous lesions were detected on both scans for all patients. Representative cases are shown in Figs 1–3.

**Fig 4:**
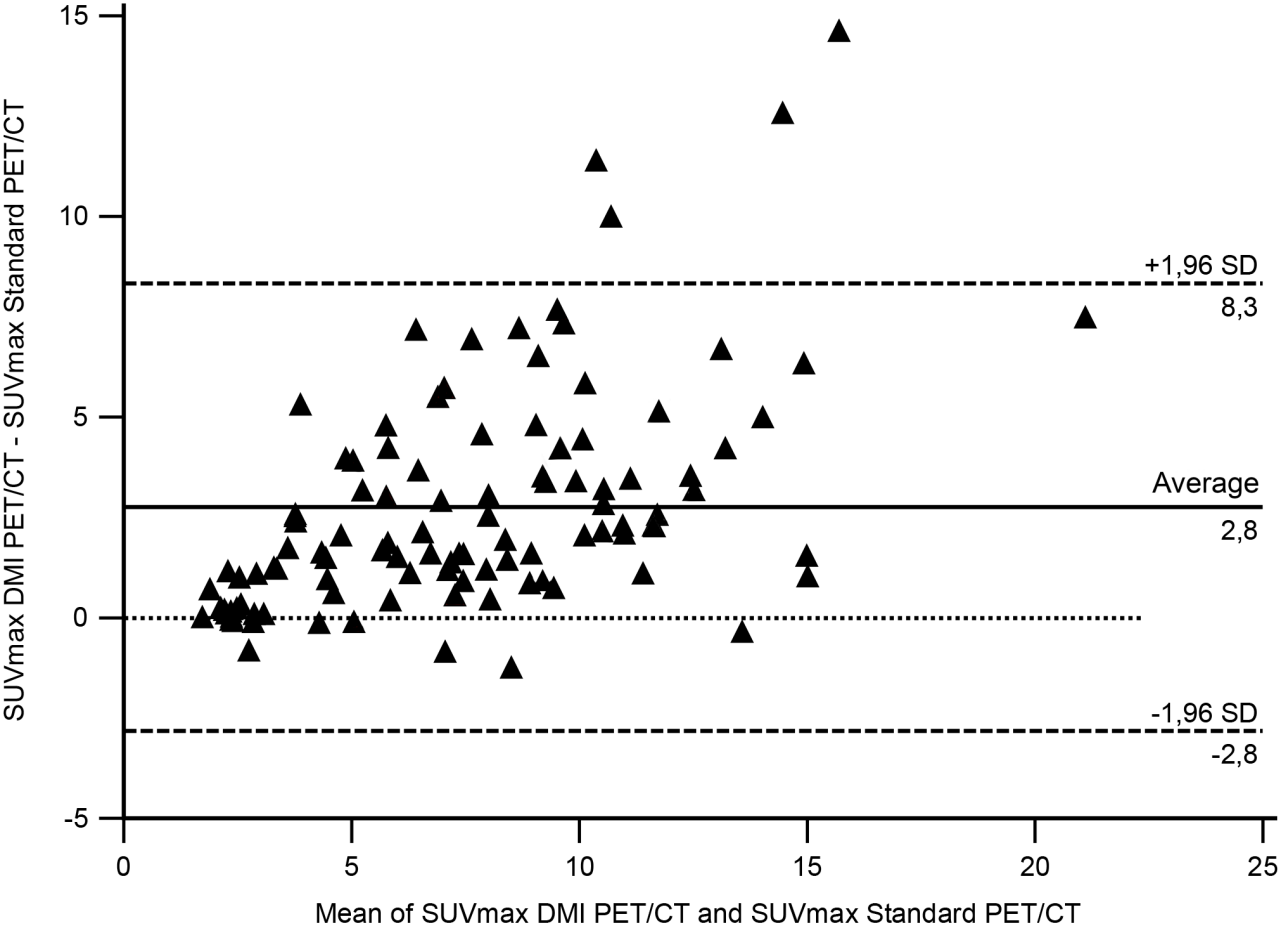
Bland-Altman plot analysis. Bland-Altman plot analysis showed an acceptable agreement between scanners for most of the SUV_max_ values registered. There is a significant bias between measurements corresponding to the higher sensitivity of DMI PET/CT. The disagreement between measurements above the 95% CI is related to lesions for which DMI PET/CT registered the greatest increase of SUV_max_.

On a per lesion analysis a total of 144 lesions were detected, of which 37 only by DMI PET/CT. Within 107 lesions detected by both scans there were 55 lymph nodes, 15 lung nodules, 9 liver lesions, 10 bone lesions and 18 other lesions (8 soft tissue lesions, 1 colon lesion, 3 head and neck lesions, 2 adrenal gland masses, 2 gastric lesions, 1 kidney and 1 spleen lesion). The agreement between lesion SUV_max_ concordance is graphically depicted with a Bland-Altman Plot in Fig 4.

**Fig 5:**
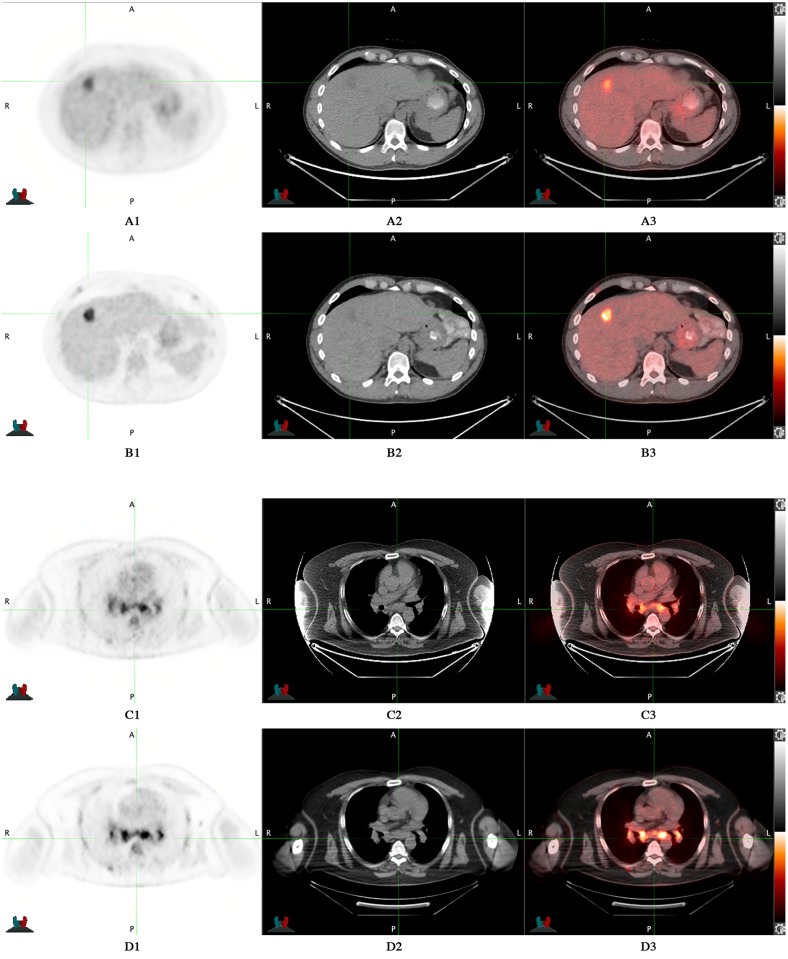
D690 PET/CT vs DMI PET/CT at different time delay between scans. A1) D690 PET; A2) D690 CT; A3) D690 fused image; B1) DMI PET; B2) DMI CT; B3) DMI fused image. Time delay between scans was 18 minutes. C1) D690 PET; C2) D690 CT; C3) D690 fused image; D1) DMI PET; D2) DMI CT; D3) DMI fused image. Time delay between scans was 73.7 minutes.

**Fig 6:**
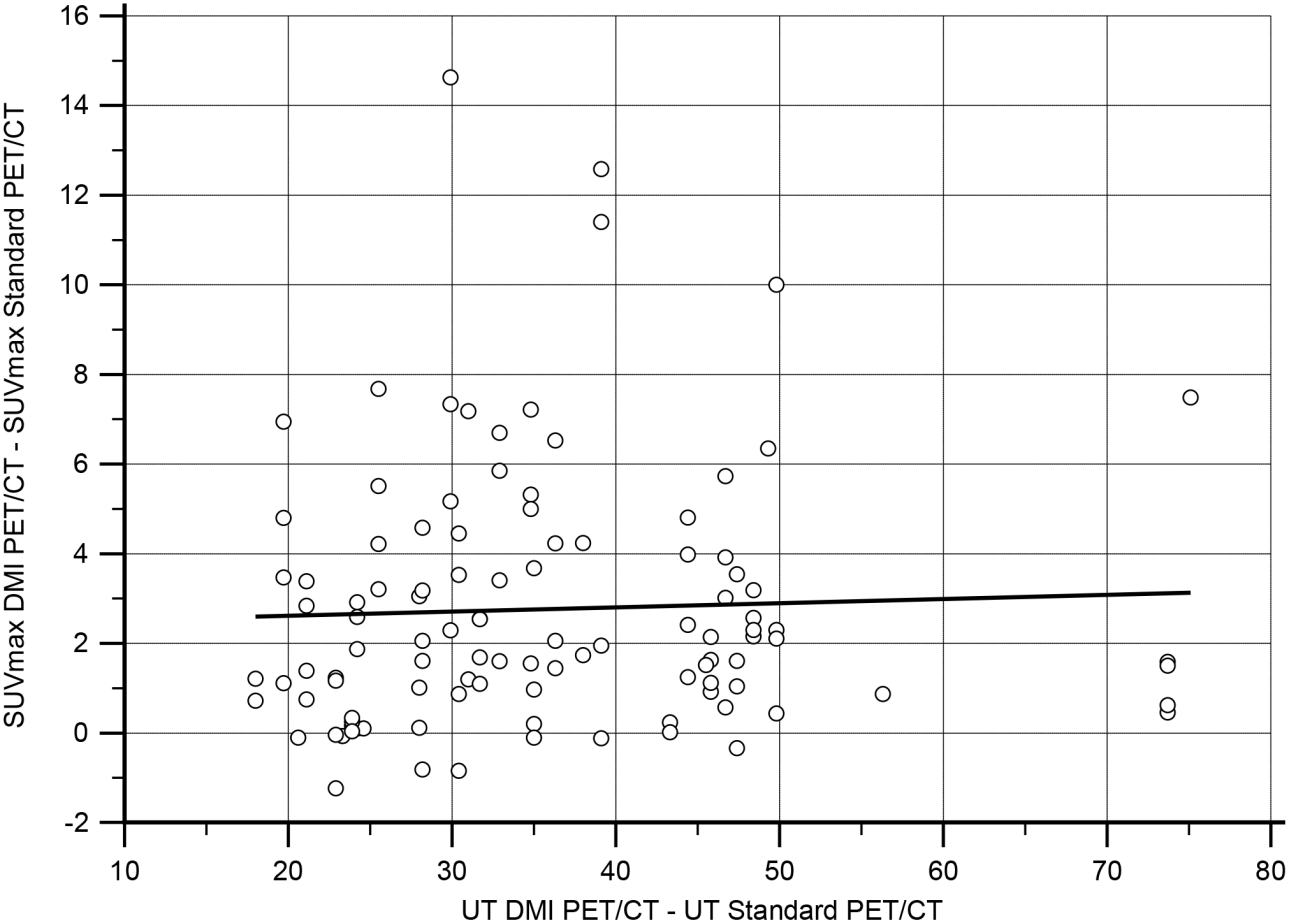
Linear regression analysis. Linear regression analysis showed no relationship between SUV_max_ and time delay. *P*-value = 0.66. UT = Uptake Time.

**Fig 7:**
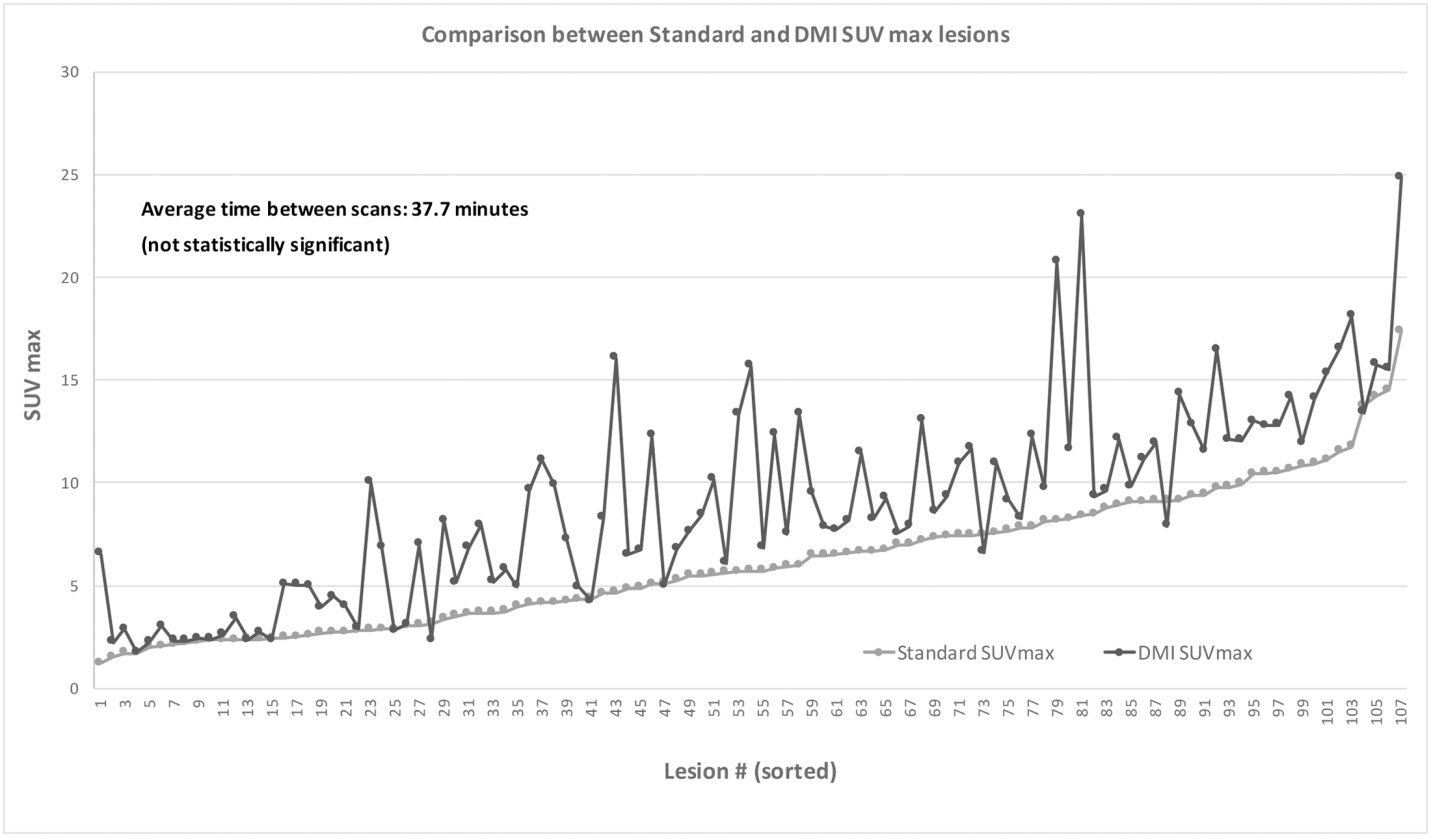
Lesional SUV_max_ measured twice over time. Recognizable increase in lesion SUV_max_ in DMI PET/CT. Partial correlation and linear regression were used to exclude time as independent variable between different SUV_max_ values.

The mean SUV_max_ were 1.2–17.4 (mean ± SD: 6.1 ± 3.3) for standard PET/CT, 1.7–24.9 (mean ± SD: 8.9 ± 4.8) for DMI PET/CT with TOF and Q.Clear^®^ and 1.7–23.6 (mean ± SD: 7 ± 4) for DMI PET/CT without TOF and Q.Clear^®^. SUV_max_ differences between standard and DMI (with TOF and Q.Clear^®^ on or off) were statistically significant for all 107 lesions (*P* < 0.0001) and not correlated to the delay to acquisition of DMI PET data (*P* < 0.0001) (Figs 5–7).

The SUV_max_ measurements for all 107 lesions were 1.2–14.6 (mean ± SD: 2.8 ± 2.8) and 3.3–7.4 (mean ± SD: 0.8 ± 1.4), higher on DMI PET/CT (TOF and Q.Clear^®^ on and TOF and Q.Clear^®^ off, respectively) compared with standard of care PET/CT.

For 14 of the 50 participants standard PET/CT used D600 (total of 30 lesions detected) and for 36 of the 50 participants it used D690 (total of 77 lesions detected). The differences stayed statistically significant (*P* < 0.05) when comparing separately differences in SUV_max_ between D600 and D690 vs DMI PET/CT (TOF and Q.Clear^®^ on or off).

The 37 lesions detected only by DMI included 24 lymph nodes, 10 lung nodules, 2 liver lesions and 1 other lesion (pleura). These additional lesions had SUV_max_ of 0.6–9.8 (mean ± SD: 3.8 ± 2.4).

The single organs analyses in 107 lesions identified on both scans showed a statistically significant difference in SUV_max_ for lymph nodes (*P* < 0.0001), lung nodules (*P* = 0.0003), bone lesions (*P* < 0.02), liver nodules (*P* < 0.01) and other lesions (*P* = 0.0004) when DMI PET images were reconstructed using TOF and Q.Clear^®^. When TOF and Q.Clear^®^ were not used, the differences in SUV_max_ between standard PET/CT and DMI PET/CT remained statistically significant for lymph nodes (*P* = 0.0004), lung nodules (*P* = 0.02), bone lesions (*P* = 0.04) and other lesions (*P* = 0.002), but not for liver (*P* = 0.16). This data is shown in Tables 2 and 3.

**Table 2:** SUV_max_ measurements in all lesions and in single organs obtained from D600/D690 and DMI PET/CT (using TOF and Q.Clear^®^).

Lesions	D600/D690 PET/CT	DMI PET/CT	Change	*P*
**All lesions (107)**	6.2±3.4	14.7 ±2.4	4.6±2.9	<0.0001
**Lymph nodes (55)**	5.6±3.3	8±4.7	2.4±3.1	<0.0001
**Lung nodules (15)**	5.6±3.6	8±4.5	2.5±2	=0.0003
**Bone lesions (10)**	8.3±3.3	12.5±5.4	4.2±4.3	<0.02
**Liver nodules (9)**	7.1±3.4	8.9±3.4	1.8±1.6	<0.01
**Other lesions (18)**	6.9±3.2	9.8±4.7	2.8±2.7	=0.004

**Table 3:** SUV_max_ measurements in all lesions and in single organs obtained from D600/D690 and DMI PET/CT (without TOF and Q.Clear^®^).

Lesions	D600/D690 PET/CT	DMI PET/CT	Change	*P*
**All lesions (107)**	6.2±3.4	6.9 ±4	0.8±1.4	<0.0001
**Lymph nodes (55)**	5.6±3.3	6.2±3.8	0.7±1.4	=0.0004
**Lung nodules (15)**	5.6±3.6	6.4±4.1	0.8±1.3	=0.02
**Bone lesions (10)**	8.3±3.3	10.1±4.3	1.8±2.4	=0.04
**Liver nodules (9)**	7.1±3.4	7.8±3.6	0.7±1.3	=0.16
**Other lesions (18)**	6.9±3.2	7.6±3.5	0.6±0.8	=0.002

We also analyzed the ratios of uptake in lesions (*n* = 107) and backgrounds tissues such as the aortic arch and liver. The lesion:aortic arch ratios ranged 0.6–11.8 (mean ± SD: 3.5 ± 2.2) for standard PET/CT and 1.3–20.5 (mean ± SD: 6.7 ± 4.2) for DMI PET/CT (*P* < 0.0001). The lesion:liver ratios ranged 0.4–8.9 (mean ± SD: 2.6 ± 1.6) for standard PET/CT and 0.8–12.2 (mean ± SD: 4.4 ± 2.5) for DMI PET/CT (*P* < 0.0001). The mean lesion:aortic arch SUV_max_ ratio and mean lesion:liver SUV_max_ ratio were 0.2–15.2 (mean ± SD: 3.2 ± 2.6) and 0.2–8.5 (mean ± SD: 1.9 ± 1.4), respectively, higher in DMI PET/CT than standard PET/CT.

A comparison of National Electrical Manufacturers Association (NEMA) testing results for D600, D690 and DMI is shown in Table 4.

**Table 4:** Comparison of NEMA testing results for D600, D690 and DMI.

	Transverse FWHM		Axial FWHM		Sensitivity (average)	Scatter fraction	Hot contrast				Cold contrast	
	1 cm	10 cm	1 cm	10 cm			10 mm	13 mm	17 mm	22 mm	28 mm	37 mm
D600 [13]	4.9 mm	5.6 mm	5.6 mm	6.4 mm	9.6 cps/kBq	36.6%	41	51	62	73	68	72
D690 [14]	4.7 mm	5.06 mm	4.74 mm	5.55 mm	7.5 cps/kBq	37%	43.8*	62.9*	70.6*	76.4*	77.3*	81.6*
DMI [15]	4.0 mm	4.01 mm	4.00 mm	5.28 mm	13.9 cps/kBq	40.43%	51.7*	61.5*	66.2*	81.3*	86.6*	90*

* Using time of flight and point spread function for data reconstruction

## Discussion

The meaningful role of hybrid imaging PET/CT in oncology has strongly been demonstrated [16]. Information provided by PET is extremely important giving a functional and metabolic assessment of normal tissue as well as disease conditions [17]. Hybrid PET/CT scanners have markedly improved in terms of sensitivity, spatial resolution and imaging reconstruction, allowing more accurate diagnoses and staging and leading to better therapeutic strategies.

In this study we have shown the potentialities of a new generation PET/CT scanner (DMI PET/CT) compared to standard of care PET/CT (D600 and D690 PET/CT) in a cohort of 50 patients. DMI PET/CT results are at least comparable to D600 and D690 in terms of lesion detectability, with DMI PET/CT identifying all the 107 lesions found using standard of care PET/CT.

The DMI PET/CT scanner uses small lutetium-based scintillator crystal arrays combined with a silicon photomultiplier (SiPM) bloc design, leading to a high NEMA sensitivity [15]. The SiPMs in DMI PET/CT enable direct conversion of photons into a digital signal that eliminates the signal loss and noise, making the scanner more efficient. In addition, the DMI PET/CT brings together the high detectors sensitivity with superior image reconstruction technology, including TOF and Q.Clear^®^. This results in increased spatial and contrast resolution, potentially allowing for the detection of smaller lesions and more accurate quantification of uptake.

Our semi-quantitative analysis showed an increase of 53.3% in SUV_max_ measurements from standard PET/CT to DMI PET/CT for all 107 lesions detected by both scanners; this difference was statistically significant and not correlated with the time delay. Similar results were assessed by Nguyen et al [18], who evaluated a prototype of digital technology PET/CT scanner and found a 36% increase in SUV_max_ in 52 avid FDG lesions from standard PET/CT to digital PET/CT.

SUV_max_ differences per single organ were statistically significant comparing standard PET/CT to DMI PET/CT with TOF and Q.Clear^®^ on; when DMI images were not reconstructed using TOF and Q.Clear^®^, differences in SUV_max_ between standard and DMI were statistically significant for all organs except the liver. We also compared separately D600 (non TOF enabled) vs DMI and D690 (TOF capable) vs DMI. DMI results (with TOF and Q.Clear^®^ on or off) were superior to standard of care PET/CT, independent to the type of scanner used; equivalence between D600 and D690 was already shown [19].

It is known that time delay can improve the diagnostic specificity of ^18^F-FDG PET/CT for tumor detection [20]. Comparing our results with previous dual-time-point studies [21, 22] we registered a higher metabolic change (53.3% vs 22.5% and 21.9%, respectively) despite a shorter time delay (37.7 vs 60 minutes), suggesting that the observed metabolic increase can be partially attributed to the better performance of DMI PET/CT.

DMI PET/CT detected 37 more areas of focal increased ^18^F-FDG uptake compared to standard of care PET/CT in 14 of 50 patients. These findings are aligned to results showed by Nguyen et al [18] who identified 8 additional ^18^F-FDG avid lesions comparing digital to standard PET/CT in 5 of 21 patients.

The higher sensitivity provided by the DMI scanner is likely to allow for reduction of the injected dose of radiopharmaceutical without an impact on image quality. It is worth noting that 8 of the 37 areas of focal increased ^18^F-FDG uptake seen only by DMI PET/CT had a diameter < 0.7 cm and mean SUV_max_ of 0.6–4.9 (mean ± SD: 2.6 ± 2). These findings suggest better small lesion detectability using DMI PET/CT compared to standard of care PET/CT.

In our study, DMI PET/CT had better lesion conspicuity as indicated by higher lesion-to-background ratios compared to standard of care PET/CT for the lesion-to-aortic arch and lesion-to-liver.

We also demonstrated the similarity of background FDG uptake between scanner types using an equivalence testing and empirically choosing ± 0.7 g/mL SUV measurement to define non-equivalent with a *P* value < 0.05. As demonstrated in Table 2, the equivalence criterion was met for all baseline organs with the exception of the cerebellum.

Our study had some limitations: the relatively small sample size of participants and the lack of histopathological confirmation for foci of ^18^F-FDG detected only by DMI PET/CT. Furthermore, we could not randomize the order of the standard PET/CT and DMI PET/CT, due to DMI pending FDA approval for clinical use at the time of the study.

Further studies in a larger cohort of patients and with histopathology as reference standard are needed to confirm these preliminary data.

## Conclusions

Discovery Molecular Insights is a new generation PET/CT scanner that brings together the sensitivity of SiPM detectors and innovative reconstruction technology. Our pilot study demonstrated that DMI PET/CT is more sensitive and can better detect smaller lesions compared to standard of care PET/CT scanners. It may make possible a reduction of the injected dose or the scan time (or a combination of both) without a reduction in image quality. Further studies will confirm its performance and its potential role in helping clinicians to diagnose and stage disease earlier and to better guide treatment strategies.

## Supporting information

S1 Dataset Anonymized data from Standard PET-CT and DMI PET-CT. (XLS)

S1 Fig Whole body (WB) standard vs WB DMI PET/CT: Background FDG uptake is similar between scanner types. A) Standard Maximum-intensity-projection (MIP) B) DMI MIP C) Standard coronal fused image D) DMI coronal fused image.
(TIF)

S2 Fig D690 PET/CT vs DMI PET/CT: DMI showed a significant increase in SUV_max_ (black arrow). DMI images were acquired 49.8 minutes after standard acquisition and 96.3 minutes after ^18^F-FDG injection. A1) D690 PET; A2) D690 CT; A3) D690 fused image B1) DMI PET; B2) DMI CT; B3) DMI fused image.
(TIF)

S3 Fig Standard vs DMI PET/CT: DMI detected an additional lymph node on the right internal mammary chain (arrow). A1) Standard PET, axial view B1) Standard CT, axial view C1) Standard fused image, axial view A2) DMI PET, axial view B2) DMI CT, axial view C2) DMI fused image, axial view.
(TIF)

S1 Table Patients’ clinical characteristics. M = male; F = female; DLBCL = diffuse large B-cell lymphoma; HL = Hodgkin lymphoma; NSCLC = non-small cell lung cancer; HCC = Hepatocellular carcinoma; FL = follicular lymphoma; UP = unknown primary.
(DOCX)
