# Supplementary figures and images for: 18F-FDG silicon photomultiplier PET/CT: A pilot study comparing semi-quantitative measurements with standard PET/CT
Source: PLoS One. 2017 Jun 5;12(6):e0178936. doi: 10.1371/journal.pone.0178936 (PMC5459477; doi:10.1371/journal.pone.0178936)

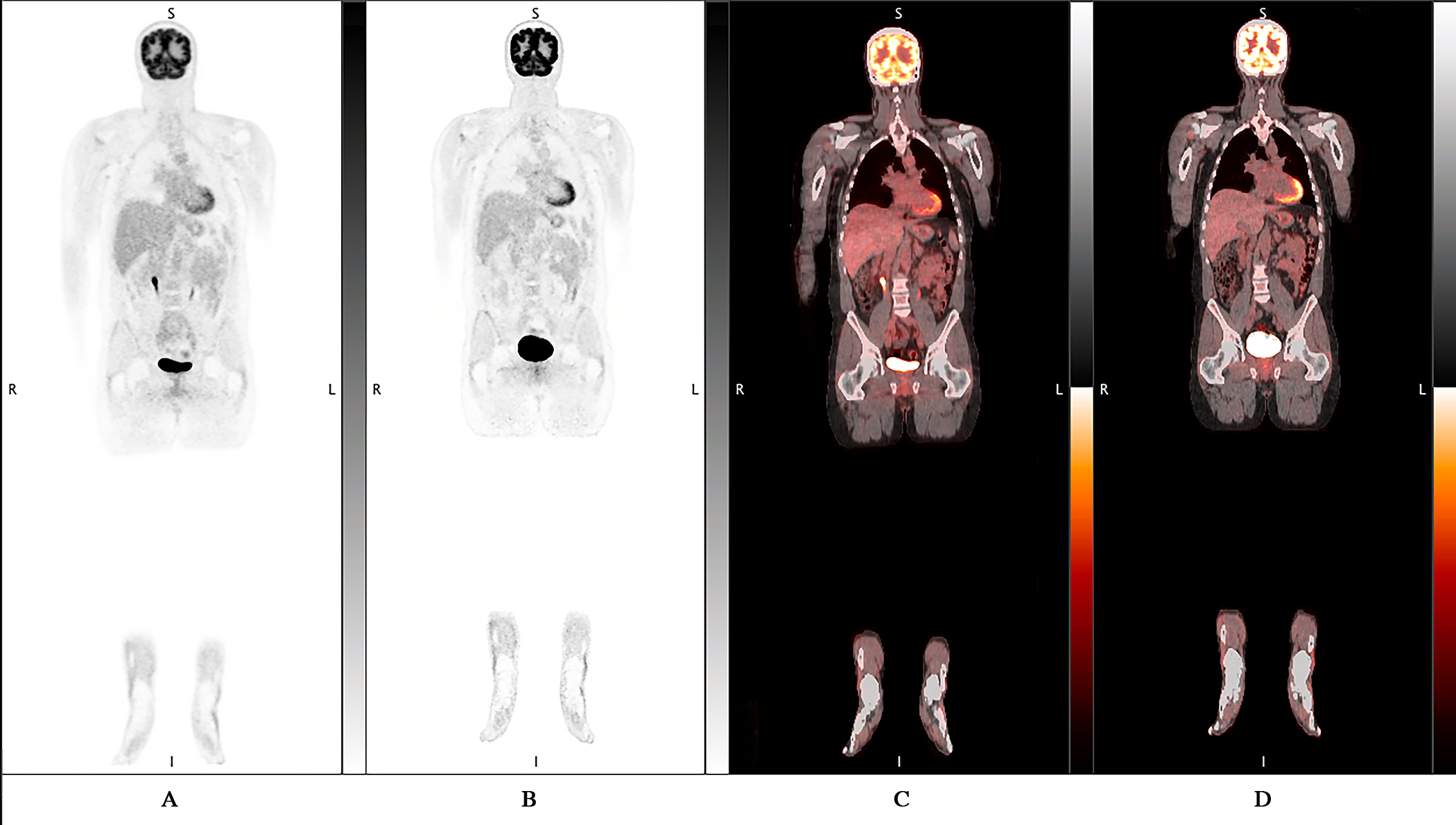

Supplement: S1 Fig — A) Standard Maximum-intensity-projection (MIP) B) DMI MIP C) Standard coronal fused image D) DMI coronal fused image. (TIF) [file pone.0178936.s002.tif]

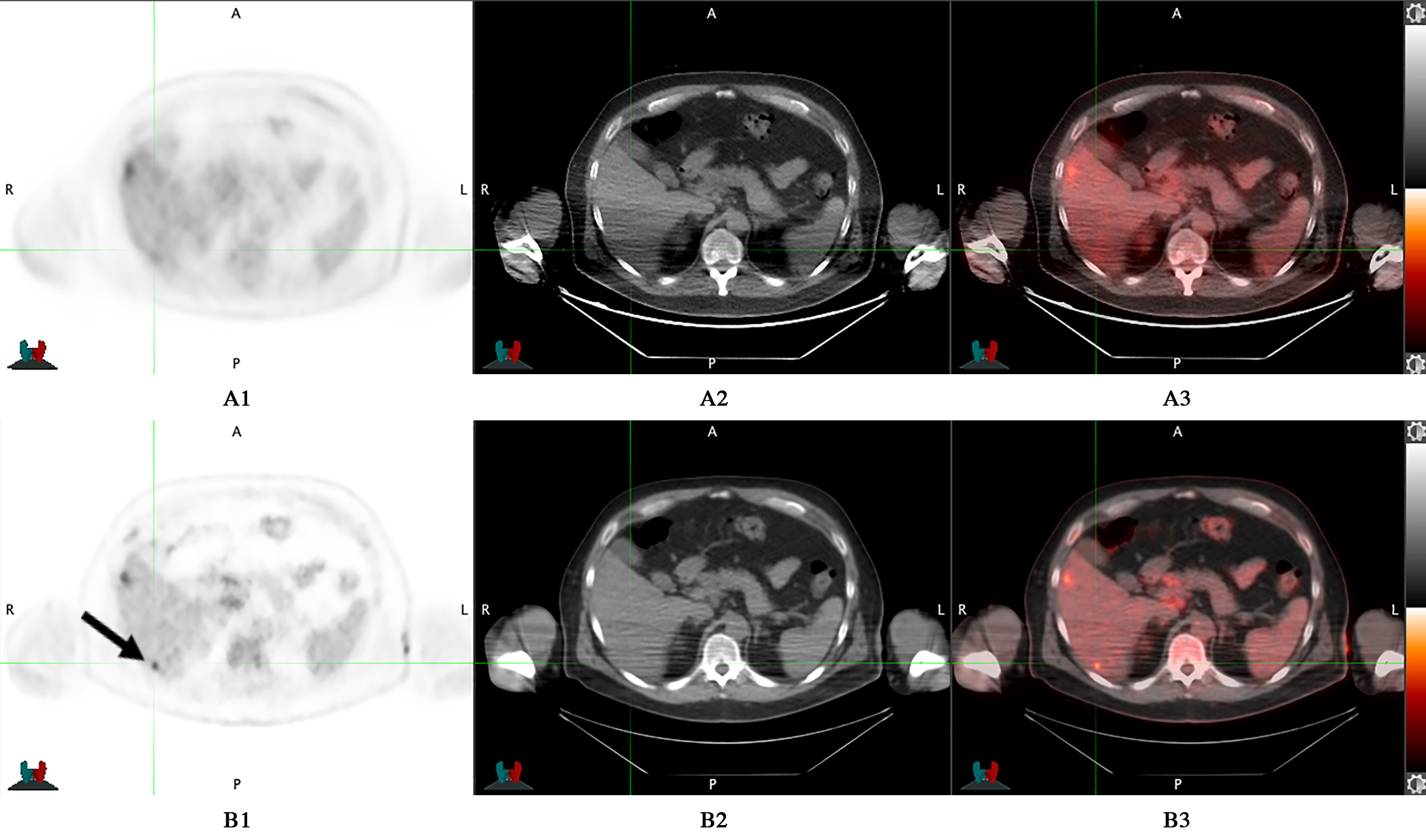

Supplement: S2 Fig — DMI images were acquired 49.8 minutes after standard acquisition and 96.3 minutes after 18F-FDG injection. A1) D690 PET; A2) D690 CT; A3) D690 fused image B1) DMI PET; B2) DMI CT; B3) DMI fused image. (TIF) [file pone.0178936.s003.tif]

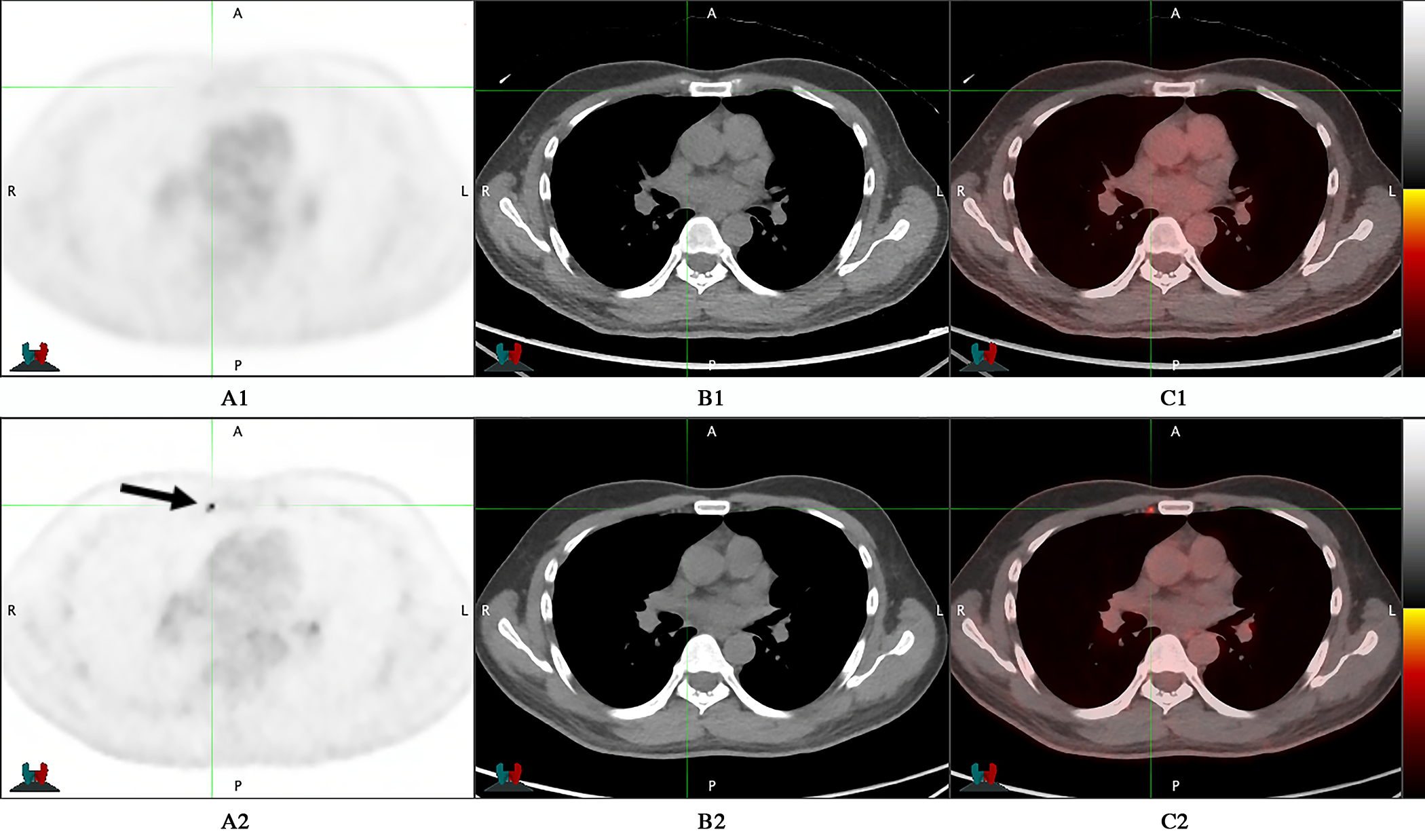

Supplement: S3 Fig — A1) Standard PET, axial view B1) Standard CT, axial view C1) Standard fused image, axial view A2) DMI PET, axial view B2) DMI CT, axial view C2) DMI fused image, axial view. (TIF) [file pone.0178936.s004.tif]
