# Supplementary material for: 18F-FDG silicon photomultiplier PET/CT: A pilot study comparing semi-quantitative measurements with standard PET/CT
Source: PLoS One. 2017 Jun 5;12(6):e0178936. doi: 10.1371/journal.pone.0178936 (PMC5459477; doi:10.1371/journal.pone.0178936)
Supplement: S1 Table — M = male; F = female; DLBCL = diffuse large B-cell lymphoma; HL = Hodgkin lymphoma; NSCLC = non-small cell lung cancer; HCC = Hepatocellular carcinoma; FL = follicular lymphoma; UP = unknown primary. (DOCX) [file pone.0178936.s005.docx]

| Patients | Age | Sex | Disease | Indication |
| --- | --- | --- | --- | --- |
| 1 | 57 | M | Colon cancer | Restaging |
| 2 | 68 | M | Melanoma | Surveillance |
| 3 | 61 | M | Tonsillar cancer | Response to therapy |
| 4 | 46 | M | Mantle cell lymphoma | Restaging |
| 5 | 77 | M | Melanoma | Response to therapy |
| 6 | 82 | M | Oropharyngeal cancer | Response to therapy |
| 7 | 59 | M | Melanoma | Response to therapy |
| 8 | 79 | M | Renal cell carcinoma | Response to therapy |
| 9 | 56 | M | Renal cell carcinoma | Response to therapy |
| 10 | 46 | M | DLBCL | Restaging |
| 11 | 46 | M | Sarcoidosis | Staging |
| 12 | 61 | M | Bladder carcinoma | Response to therapy |
| 13 | 66 | M | NSCLC | Response to therapy |
| 14 | 62 | M | Base of tongue carcinoma | Restaging |
| 15 | 37 | M | HL | Response to therapy |
| 16 | 52 | M | Melanoma | Staging |
| 17 | 53 | M | Mantle cell lymphoma | Response to therapy |
| 18 | 70 | M | Follicular lymphoma | Response to therapy |
| 19 | 72 | M | Lung cancer | Staging |
| 20 | 50 | M | HL | Staging |
| 21 | 80 | M | HL | Response to therapy |
| 22 | 27 | M | HL | Restaging |
| 23 | 63 | M | Lung cancer | Staging |
| 24 | 55 | M | Melanoma | Response to therapy |
| 25 | 53 | M | Left upper pulmonary nodule | Restaging |
| 26 | 80 | F | Breast cancer | Response to therapy |
| 27 | 69 | M | NSCLC | Response to therapy |
| 28 | 72 | F | Endometrial cancer | Restaging |
| 29 | 44 | M | Chronic lymphocytic leukemia | Staging |
| 30 | 80 | M | Angiosarcoma | Response to therapy |
| 31 | 52 | M | Base of tongue carcinoma | Response to therapy |
| 32 | 81 | M | NSCLC | Staging |
| 33 | 75 | F | NSCLC | Restaging |
| 34 | 69 | M | HCC | Surveillance |
| 35 | 72 | F | Esophageal cancer | Staging |
| 36 | 79 | F | Thyroid cancer | Restaging |
| 37 | 41 | M | Adrenocortical carcinoma | Surveillance |
| 38 | 94 | M | Gastric malignancy | Restaging |
| 39 | 56 | M | Pulmonary sarcoidosis | Staging |
| 40 | 83 | M | DLBCL | Restaging |
| 41 | 39 | M | Rectal cancer | Surveillance |
| 42 | 66 | M | Indolent FL | Response to therapy |
| 43 | 52 | M | HL | Surveillance |
| 44 | 35 | M | Testicular cancer (seminoma) | Restaging |
| 45 | 54 | M | Colon adenocarcinoma | Response to therapy |
| 46 | 34 | M | Testicular cancer | Restaging |
| 47 | 85 | M | Adenocarcinoma UP | Restaging |
| 48 | 54 | F | Breast cancer | Restaging |
| 49 | 62 | M | Colorectal cancer | Restaging |
| 50 | 42 | M | DLBCL | Response to therapy |
